# Evaluating the health economic impact of cefepime/enmetazobactam in complicated urinary tract infections in the German setting: a cost analysis from payer perspective

**DOI:** 10.1007/s15010-025-02711-9

**Published:** 2025-12-26

**Authors:** Johanna Röder, Sebastian M. Wingen-Heimann, Danila Seidel, Ann-Cathrine Froitzheim, Melina S. Kurte, Oliver Witzke, Maria J. G. T. Vehreschild, Oliver A. Cornely, Florian Kron

**Affiliations:** 1 VITIS Healthcare Group, Cologne, Germany; 2 Institute of Translational Research, Cologne Excellence Cluster on Cellular Stress Responses in Aging-Associated Diseases (CECAD), Faculty of Medicine, University Hospital Cologne, University of Cologne, Cologne, Germany; 3 Department I of Internal Medicine, Faculty of Medicine and University Hospital Cologne, Center for Integrated Oncology Aachen Bonn Cologne Duesseldorf (CIO ABCD, Excellence Centre for Medical Mycology (ECMM), University of Cologne, Cologne, Germany; 4 German Center for Infection Research (DZIF), Partner Site Bonn-Cologne, Cologne, Germany; 5 Faculty of Medicine, University Duisburg-Essen, Essen, Germany; 6 Department of Infectious Diseases, West German Centre of Infectious Diseases, University Hospital Essen, University Duisburg-Essen, Essen, Germany; 7 Department II of Internal Medicine, Infectious Diseases, Goethe University Frankfurt, University Hospital Frankfurt, Frankfurt Am Main, Germany; 8 Fraunhofer Institute for Translational Medicine and Pharmacology ITMP, Frankfurt Am Main, Germany; 9 Department I of Internal Medicine/Center for Integrated Oncology (CIO Cologne), Faculty of Medicine and University Hospital Cologne, University of Cologne, Cologne, Germany; 10 FOM University of Applied Sciences, Herkulesstraße 32, 45127 Essen, Germany

**Keywords:** cUTI, Health economic analysis, Cefepime/enmetazobactam, Piperacillin/tazobactam

## Abstract

**Objectives:**

Complicated urinary tract infections (cUTIs) are ubiquitous, associated with healthcare resources, and demand effective antibiotic treatment to prevent clinical failure and relapse. Evidence on health economic implications of treatment options remains limited. Recent studies have shown that cefepime/enmetazobactam was superior to piperacillin/tazobactam regarding the combined endpoints clinical cure and microbiological eradication, the latter being closely linked to reduced relapse rates. Therefore, we perform a health economic evaluation of cefepime/enmetazobactam vs. piperacillin/tazobactam for cUTI from a German payer perspective.

**Methods:**

To assess monetary impacts of both therapies, we conducted a semi-structured literature review for costs of (relapsed) cUTI. Subsequently, we adjusted international costs to the German healthcare system using European price levels of the Organisation for Economic Cooperation and Development. These built the basis of a comparative health economic analysis using a decision tree incorporating outcome probabilities and relapse rates for both antibiotics. Lastly, we validated the analysis using publicly available remuneration data from German hospitals.

**Results:**

Literature revealed international costs of €5,394 and €6,675 per patient without and with clinical relapse, converting to €5,137.14 and €6,357.14 in Germany, respectively. Considering the probability of occurrence of clinical cure, microbiological persistence, and relapse rates, average treatment costs per patient for cefepime/enmetazobactam amount to €5,332.12 compared to €5,414.83 for piperacillin/tazobactam.

**Conclusion:**

The analysis shows that a higher probability of relapse after antibiotic therapy might be associated with an increase in treatment costs within the German healthcare system. Although per-patient cost differences between cefepime/enmetazobactam and piperacillin/tazobactam are moderate, their cumulative impact at the population level could be substantial, emphasizing the broader health-economic relevance of treatment choice for cUTI.

**Supplementary Information:**

The online version contains supplementary material available at 10.1007/s15010-025-02711-9.

## Introduction

Urinary tract infections (UTI) are among the most common bacterial infections and represent a major public health concern due to their high prevalence and significant impact on healthcare systems worldwide [1, 2, 3]. Particularly complicated urinary tract infections (cUTI), which are characterised by occurring in patients with structural or functional anomalies of the urogenital tract [4], or in combination with comorbidities, result in higher healthcare costs. However, limited research exists regarding the extent of the clinical and financial burden [1, 5, 6, 7]. Broad definitions and delimitations of cUTI limit comparability of studies, especially on an international scale [8]. Direct and indirect costs associated with cUTI arise from prolonged hospital stays, illness-related disability, additional diagnostic procedures, and the need for prolonged antimicrobial therapy [5]. These costs are further enhanced by emergence of multidrug-resistant bacterial pathogens, making cUTI treatment more difficult [2, 6, 9]. It is not uncommon for cUTIs to occur during an inpatient stay and, at up to 65%, they account for the largest proportion of nosocomial infections [5, 10]. Additionally, cUTI represent a reason for admission to hospital [11]. A cross-sectional multicenter study based on the National Emergency Department database in the United States has shown that almost two thirds of cUTI visits in the emergency department ended in hospitalisation. Of these, 44.9% were related to cUTI directly as main diagnosis, whereas 85.5% had cUTI as secondary diagnosis [12]. In light of these challenges, there is an urgent need for treatment strategies to address resistance but also to reduce relapse rates and, consequently, healthcare resource utilisation. However, health economic evaluations for this purpose are rare and not yet available for cUTI in Germany.

Clinical trial data have demonstrated that the novel *β*-lactam/*β*-lactamase inhibitor combination cefepime/enmetazobactam is non-inferior and superior in efficacy to piperacillin/tazobactam for treating cUTI and acute pyelonephritis caused by gram-negative bacteria [13]. The combined measure of clinical cure and microbiological eradication showed that more patients achieved favourable outcomes with cefepime/enmetazobactam. Microbiological eradication was defined as the reduction of qualifying baseline pathogens to less than 10^3^ CFU/mL in urine [13]. The composite outcome of both clinical cure and microbiological eradication to determine therapeutic success is commonly used by the US Food and Drug Administration (FDA) and the European Medicines Agency (EMA) [27, 28]. Kadry et al. showed that microbiological eradication in particular is an indicator of the overall success of the antibiotic therapy, i.e. also considering relapse rates [14]. Patients who met the criteria for clinical cure without microbiological eradication at follow-up after completing antibiotic therapy were with an adjusted odds ratio of 5.51 more likely to experience clinical failure compared to those who achieved both clinical cure and microbiological eradication [14].

In this study, the health economic impact of using cefepime/enmetazobactam instead of piperacillin/tazobactam for the treatment of cUTI is evaluated from a payer’s perspective, with a particular focus on reducing relapse rates and hospital readmissions, and the subsequent impact on healthcare resource utilisation and costs in Germany.

## Methods

The following step-by-step approach was used for the health economic evaluation to assess the monetary impact for the treatment of cUTI patients with cefepime/enmetazobactam:

I.Cost evaluation for cUTI The cost assessment for cUTI in Germany was conducted based on a semi-structured literature review by using the literature database PubMed. Relevant sources were analysed to estimate the average cost per patient on an inpatient level.

II.Cost evaluation for relapse of cUTI in Germany The costs associated with relapses of cUTI were also derived from a semi-structured literature research with PubMed, followed by a conversion of costs into the German healthcare context via an international comparison of price levels of the Organisation for Economic Cooperation and Development (OECD).

III. Comparative health economic analysis of cefepime/enmetazobactam vs. piperacillin/tazobactam for cUTI in Germany The results from step I and step II served as the foundation for the comparative health economic evaluation for the two antibiotic regimes cefepime/enmetazobactam versus piperacillin/tazobactam for the treatment of cUTI. The comparison accounted for the cUTI relapse rate, incorporating primary outcome and recurrence rates as reported in previous studies [13, 14]. By integrating these probabilities into the cost framework, a comprehensive assessment of the total healthcare expenditures associated with each treatment strategy was performed from a healthcare payer’s perspective.

IV.Validation of simulated cost data in Germany To ensure the robustness of the cost estimates, a final verification step was conducted using publicly available inpatient data from all German hospitals, completed by cost data from cost-accounting hospitals for the year 2023. According to § 21 Hospital Remuneration Act (Krankenhausentgeltgesetz (KHEntG) inpatient case data is reported quarterly to the Institute for Hospital Remuneration system (InEK). The data contained case information on diagnoses, procedures and outsourced German Diagnosis Related groups (aG-DRGs) in an anonymised and aggregated form and is accessible via the database InEK Data Browser [15]. Twenty-five queries consisting of principal diagnosis and secondary diagnoses (Supplementary Table 8) based on the inclusion criteria of Kaye et al. [13] were performed, to create an equivalent study (queries 1-25). Procedural codes (OPS) for complex treatment for colonisation or infection with multidrug-resistant pathogens were further queried (Supplementary Table 9). Another query (query 26) combines the main diagnosis for urinary tract infections (N39.0) according to ICD-10-GM with relevant secondary diagnoses and an ICD specific multidrug-resistant pathogens. Supplementary Table 10 shows the composition of all 26 performed queries, including all diagnostic and procedure codes according to the International Statistical Classification of diseases 10 German Modification (ICD-10-GM) 2023 [16] and the German procedure classification system 2023 [17].

### Data collection

The direct cost data underlying this study was obtained from Vallejo-Torres et al. [18] and consisted of unit costs for length of stay (LOS), diagnostic tests (DIAG), treatment procedures (TREAT), antibiotic therapy (ATB), and costs after discharge for readmissions and outpatient visits (DISCH). The study presented three cost scenarios for cUTI patients. Scenario 1 includes costs for diagnosis and treatment, and the length of stay in the hospital. The second scenario considers in addition the costs of antibiotic therapy. The third scenario furthermore considers costs incurred after the actual discharge with respect to readmissions and additional outpatient visits after relapse. The scenarios two and three were used for the following cost analysis and transferred to the German healthcare system.

### International comparison of healthcare values

For international comparison of healthcare values, a methodology developed by the OECD was used. Because price levels vary markedly between Germany, Spain, and other European countries, Koechlin et al. [19] and Lorenzoni et al. [20] developed an approach of converting national currency values into one common European price level. For this study, the Spanish cost values for cUTI treatment as published by Vallejo-Torres et al. [18] were converted to German cost values via the common European price level as presented in Table 1. This applied methodology ensures the transferability of cost values to the German healthcare system.

**Table 1:** Cost value conversion Spain–Germany for three scenarios based on OECD price level

	Spain [18]	OECD-Level 2017	Germany
LOS + DIAG + TREAT	€5,241.00	€6,239.29	€4,991.43
LOS + DIAG + TREAT + ATB	€5,394.00	€6,421.43	**€5,137.14**
LOS + DIAG + TREAT + ATB + DISCH	€6,675.00	€7,946.43	**€6,357.14**

Bold values indicate the resulng costs used for the decision tree

LOS - length of stay, DIAG - diagnostic tests, TREAT - treatment procedures, ATB - antibiotic therapy, DISCH - costs after discharge for readmissions and outpatient visits

Spain’s healthcare price level is 84% of the OECD average, whereas Germany’s is 80%. For example, €100 spent on healthcare services in Spain corresponds to €95.24 in Germany when adjusted for OECD price levels

### Decision tree

For the comparative cost analysis for cUTI in Germany, a decision tree model was constructed. Literature-based and converted cost values (Table 1) were incorporated as well as probabilities for “clinical cure AND microbiological eradication” (composite endpoint), probabilities for “clinical cure AND microbiological persistence”, probabilities for “No clinical cure AND microbiological persistence” (Table 2) from Kaye et al. [13], and probabilities for “clinical relapse” and “no relapse/readmission” (Table 3) from Kadry et al. [14] to calculate overall cUTI treatment costs for cefepime/enmetazobactam versus piperacillin/tazobactam. Relapse rates are conditional on microbiological outcome and were applied equally to both drugs due to lack of drug-specific evidence [14].

**Table 2:** Probabilities I for decision-tree based on Kaye et al. [13]

	Clinical cure AND microbiological eradication	Clinical cure AND microbiological persistence	No clinical cure AND microbiological persistence	*∑*
Cefepime/enmetazobactam	79.1%	13.4%	7.5%	100%
Piperacillin/tazobactam	58.9%	30.0%	11.1%	100%

**Table 3:** Probabilities II for decision tree based on Kadry et al. [14]

	Clinical relapse/readmission	No relapse/readmission	*∑*
Clinical cure AND microbiological eradication	6.2%	93.8%	100%
Clinical cure AND microbiological persistence	26.7%	73.3%	100%
No clinical cure AND microbiological persistence	100%	0%	100%

### Sensitivity analysis

Sensitivity analysis to control for variation in probabilities was conducted based on the approach by Kadry et al. [14]. Supplementary Table 11 shows the modified probabilities used for the sensitivity analysis.

## Results

### Decision tree

**Fig. 1:**
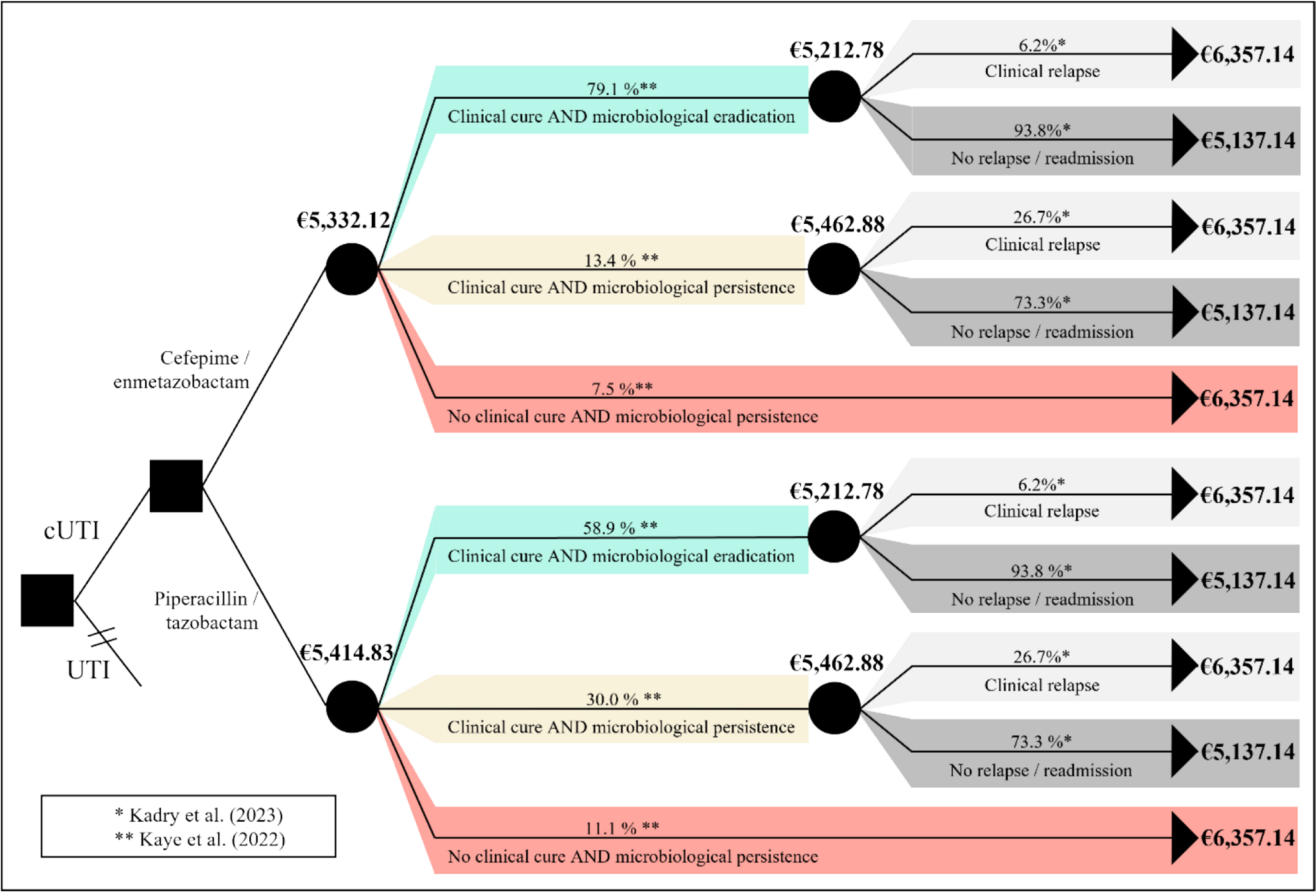
Decision Tree cUTI treatment and associated costs with cefepime/enmetazobactam vs. piperacillin/tazobactam. Relapse rates are conditional on microbiological outcome and were applied to both drugs due to lack of drug-specific evidence

For Spain, cost values of €5,394 without a clinical relapse and €6,675 with a clinical relapse for the treatment of cUTI were reported in the literature [18]. The conversion using the OECD price levels of 84% for Spain and 80% for Germany resulted in costs of €5,137.14 for the treatment of cUTI without a relapse and €6,357.14 for cases with a relapse in Germany (Fig. 1, calculations in Supplementary Table 7). The resulting costs for cases with clinical relapse and those without relapse were assumed to be independent of the selected antibiotic therapy in this model, as published literature does not provide robust evidence for differences in relapse-related costs between cefepime/enmetazobactam and piperacillin/tazobactam. Considering the probabilities of clinical relapse and non-relapse, this results in the following costs for the two different scenarios cefepime/enmetazobactam and piperacillin/tazobactam of the decision tree model. To determine the costs to be compared, the decision tree must be viewed from right to left; the total costs result from the multiplication of case costs with the respective probabilities.

#### Cefepime/enmetazobactam

In patients who undergo treatment with cefepime/enmetazobactam, clinical cure AND microbiological eradication is reached with a probability of 79.1%. Assuming that a clinical relapse occurs in 6.2% and no relapse in 93.8% of these cases, this results in average composed costs for therapy of €5,212.78 for the composite endpoint. Clinical cure AND microbiological persistence are achieved in 13.4% of cases, with the assumption of clinical relapse occurring with a probability of 26.7% and non-relapse with a probability of 73.3%. This results in average treatment costs of €5,462.88. However, 7.5% of all cases treated with cefepime/enmetazobactam did not meet clinical cure nor microbiological eradication, resulting in treatment costs of €6,357.14 within this group with a probability of 100%. This results in average treatment costs of €5,332.12 for cefepime/enmetazobactam.

#### Piperacillin/tazobactam

Patients treated with the comparator piperacillin/tazobactam reach the composite endpoint clinical cure AND microbiological eradication in 58.9% of cases. Probabilities for the assumption of clinical relapse (6.2%) and no relapse (93.8%) remain equal, resulting in average composed costs of €5,212.78. Clinical cure AND microbiological persistence are observed in 30.0% of all cases with treatment of piperacillin/tazobactam. Probabilities of 26.7% for clinical relapse and 73.3% for no relapse result in average costs of €5.462,88. No clinical cure AND microbiological persistence is met by 11.1% of patients treated with piperacillin/tazobactam, equally to treatment costs of €6,357.14 for this group. Overall, average treatment costs for piperacillin/tazobactam amount to €5,414.83 without further differentiation of clinical relapse and no relapse.

### Results verification

We performed 26 individual queries via the InEK DataBrowser to obtain data for the German healthcare setting. The resulting DRGs were matched with the average costs per DRG from the InEK DataBrowser—Data Delivery Costs DRG 2023 grouped by 2025 (DRG Report Browser). The queries can be clustered as shown below.

#### Queries 1–25

A total of 169 cases (queries 1–25) met the inclusion criteria for cUTI derived from Kaye et al. [13]. Due to data protection regulations, a number of cases smaller than four is not displayed in the InEK DataBrowser. This results in 77 cases for further analysis, whereas 92 cases were excluded. Forty of all included cases were assigned to DRG L63B with an average LOS of 13.4 days (Table 4). The remaining 37 cases were assigned to DRG L63A with an average LOS of 19.9 days (Table 5). This results in an average DRG- costs of €5,312.12 per case with DRG L63B, and €7,809.39 per case for the higher-rated DRG L63A for the accounting year 2023. Both groups were within the average length of stay of the respective DRG-group, ensuring no further surcharges or deductions from the DRG-fee. Due to the separation of the nursing budget from the DRG, personnel costs for nursing were not included.

**Table 4:** Verification—DRG costs L63B for cUTI in Germany

aG-DRG	Designation	Query No.	Cases	Average LOS	Standard deviation LOS	Cumulated LOS
L63B	Infections of the urinary organs without certain highly complex treatment, with certain complex treatment or with extremely severe CC, without complex treatment for pathogens requiring isolation, without extremely severe CC. [27]	1	11	12.00	5.69	132
		3	5	12.60	2.06	63
		4	5	16.00	10.68	80
		11	8	11.25	5.61	90
		16	11	15.55	8.50	171
			40	13.4		536
	**L63B – Arithmetic mean costs aG-DRG ** **2023 grouped by 2025**, [26]		€5,312.12			
	**Standard deviation costs aG-DRG L63A**		€2,767.51			

aG-DRG - outsourced German Diagnosis-Related Group, LOS - length of stay

**Table 5:** Verification—DRG-remuneration L63A for cUTI in Germany

aG-DRG	Designation	Query No.	Cases	Average LOS	Standard deviation LOS	Cumulated LOS
L63A	Infections of the urinary organs with certain highly complex treatment or with extremely severe CC, with complex treatment for pathogens requiring isolation. [27]	1	12	20.50	14.19	246
		4	10	19.60	9.71	196
		11	6	17.17	8.09	103
		16	9	21.44	9.62	193
			37	19.9		738
	**L63A – Arithmetic mean costs aG-DRG ** **2023 grouped by 2025** [26]		€8,686.43			
	**Standard deviation costs aG-DRG L63A**		€3,751.82			

aG-DRG - outsourced German Diagnosis-Related Group, LOS - length of stay

#### Query 26

Query 26 was based on the main diagnosis for urinary tract infections (N39.0) combined with relevant secondary diagnoses and further ICD codes for specific multidrug-resistant pathogens. We received a total of 114 cases in this query. 17 cases were excluded by data protection regulation. 46 cases (40.32%) resulted in the two previously stated DRGs L63B (32 cases) and L63A (14 cases) corresponding to average treatment costs of €5,312.12 for L63B and €8,686.43 for L63A, respectively. Four additional DRGs were found (L63E, L64B, L74Z, L44Z), associated with aG-DRG costs between €1,900.34 and €7,130.87 for the average LOS resulting from the query (Table 6).

**Table 6:** Verification query 26 for cUTI in Germany

aG-DRG	Designation	Cases	aG-Costs aG-DRG 2023 grouped by 2025	Standard deviation costs aG-DRG
L63B	Infections of the urinary organs without certain highly complex treatment, with certain complex treatment or with extremely severe CC, without complex treatment for pathogens requiring isolation or with complex treatment for pathogens requiring isolation, without extremely severe CC	32	€5,312.12	€2,767.51
L63E	Infections of the urinary organs without extremely severe CC, without moderate / complex / highly complex treatment, without complex treatment for pathogens requiring isolation. treatment, without complex treatment for pathogens requiring isolation, without certain severe infections, age >5 and <18 years, without severe CC or age >17 and <90 years	31	€2,277.84	€1,044.99
L63A	Infections of the urinary organs with certain highly complex treatment or with extremely severe CC, with complex treatment for pathogens requiring isolation	14	€8,686.43	€3,751.82
L64B	Other diseases of the urinary organs with extremely severe or severe CC or specific diagnosis, more than one day of hospitalisation or urethrocystoscopy, except for congenital malformation, age >2 years	9	€1.917,49	€783.09
L74Z	Certain diseases and disorders of the urinary organs with para-/tetraplegia	6	€2,544.50	€1,274.27
L44Z	Geriatric early rehabilitation complex treatment for diseases and disorders of the urinary organs	5	€6,546.29	€2,039.51

Arithmetic mean costs and standard deviation based on Data delivery Costs DRG 2023 grouped by 2025 (‘DRG Report Browser’) [26]

### Sensitivity analysis

The sensitivity analysis was based on the sensitivity analysis of Kadry et al. (2023) who stratified participants by reported reasons for clinical failure, resulting in different probabilities: 5.1% (compared to 6.2%) of concordant successes (clinical cure, microbiological eradication) were clinical failures defined as relapses, whereas 17.5% of discordant failures (clinical cure, microbiological persistence) resulted in relapses. Considering these probabilities, result tendency remains robust leading to average composed costs of €5,306.47 for treatment with cefepime/enmetazobactam compared to €5,373.25 for piperacillin/tazobactam treatment. Supplementary Tables 11 and 12 and Supplementary Fig. 2 show the corresponding probabilities, calculations and the resulting decision tree.

## Discussion

To our knowledge, this is the first study analysing the financial effects of cUTI with and without relapses in Germany, comparing the impact of the two antibiotics cefepime/enmetazobactam and piperacillin/tazobactam within an economic evaluation. The results of this study highlight the health economic impact of treating cUTI with cefepime/enmetazobactam compared to piperacillin/tazobactam from the payer perspective in Germany. By incorporating literature-based cost values and clinical trial probabilities for relapse rates into a decision tree, we modelled the potential healthcare resource utilisation and financial burden associated with cUTI treatment strategies.

Our analysis reveals that relapse/readmission for cUTI has a direct financial impact and leads to significant increase in healthcare costs. Based on the semi-structured literature review, the average cost of treating a cUTI without relapse/readmission is €5,137.14, compared to €6,357.14 for cUTI with relapse/readmission from a payer’s perspective in Germany. Treatment costs are also influenced by the choice of antibiotic, as differences in drug efficacy can affect the likelihood of relapse and the duration of therapy. On average, treating a cUTI with cefepime/enmetazobactam results in costs of €5,332.12, while treatment with piperacillin/tazobactam results in €82.71 higher costs, when accounting for all outcome contingencies.

These findings are generally reflected in our decision-tree model and validation. Costs for more severe cases of cUTI were estimated at €8,686.43, whereas less severe cases amounted to €5,312.12. However, while the model supports the general trends observed in the literature, these findings should be interpreted with caution, as the aggregated aG-DRG case data used for modelling did not allow for the specific distinction between clinical relapse and cases without relapse/readmission. Consequently, the resulting cost estimates may be conservative, potentially underestimating the true economic burden, as relapse-related readmissions and resulting long-stay outlier payments are not fully captured.

Although the per-patient cost difference between cefepime/enmetazobactam and piperacillin/tazobactam is relatively small, its implications may be more relevant when considered at population level. Between 2022 and 2024, approximately 240,000–280,000 patients in Germany were hospitalised annually with cUTI/otherwise-defined UTI as the main reason for admission (ICD-10-GM N30-N39, Other diseases of the urinary system) [15, 21, 22]. Assuming all were treated with cefepime/enmetazobactam instead of piperacillin/tazobactam, potential annual cost savings could range from €19.9 million to €23.1 million. These theoretical estimates represent an upper-bound scenario and should be interpreted with caution, as only a subset of patients would be eligible for treatment with a reserve antibiotic. Nonetheless, even with these constraints, these results suggest that substantial population-level savings are possible, highlighting the potential economic relevance of broader adoption in clinical practice. This consideration may become even more relevant when extrapolating to larger healthcare systems.

Previous studies have shown that microbiological persistence, even after initial clinical cure, increases the risk of later treatment failure [14]. Therefore, initiation with effective antimicrobial therapy, leading to both clinical cure and microbiological eradication, is crucial to minimize the risk of relapse and subsequent hospital readmission. Our results add an important economic dimension to the clinical perspective by showing that relapses are associated with notably higher healthcare costs. As shown in the model, the higher efficacy of cefepime/enmetazobactam in achieving both clinical and microbiological success translates into both improved patient outcomes and reduced healthcare expenditures from a payer perspective.

From a health economic perspective, our analysis highlights the importance of choosing antibiotics that not only address antimicrobial resistance but also minimize indirect costs, such as prolonged hospital stays and additional diagnostic interventions. The decision tree model provides a structured approach to quantifying these economic effects on a case basis, offering valuable insights for healthcare payers in Germany. Given the increasing prevalence of multidrug-resistant pathogens, treatment strategies that effectively reduce relapse rates can help mitigate the long-term financial burden on the healthcare system.

It should be noted that our analysis set its focus on quantifying the economic burden for the treatment of cefepime/enmetazobactam compared to piperacillin/tazobactam broken down to a case-based level for Germany. Hereby, the focus is set on cUTI as the main diagnosis. However, cUTI as a secondary diagnosis and hospital-acquired infection poses a significant share as demonstrated by Zilberberg et al. [12]. Due to the heterogeneity of cUTI cases and limitations of the InEK Data Browser as data source, it was not possible to derive an overall German picture from our aggregated data source. The expansion of the analysis to cover the broader cUTI population should be addressed in future analyses.

Finally, it needs to be stressed that the favourable evaluation of cefepime/enmetazobactam compared to piperacillin/tazobactam with respect to health economic aspects is only one of many factors that need to be taken into account in a clinical context. From an antimicrobial stewardship perspective [23], the replacement of piperacillin/tazobactam by cefepime/enmetazobactam in the absence of multidrug-resistant bacteria is not acceptable and needs to be taken into account during further discussions. In this line of thought, it would be very valuable to extend the health economic analysis to further drugs with a comparable activity spectrum.

### Limitations

This study has several limitations. The model relies on probabilities from Kaye et al. [13], who predominantly enrolled patients from Eastern Europe. Eastern Europe is a region that is assumed to have comparatively higher rates of ESBL-producing pathogens than Western Europe [24, 25], which may have influenced treatment outcomes. In the study by Kadry et al. [14], relapse rates were primarily assessed at end of treatment, whereas our evaluation focused on day 14 outcomes, when intergroup differences were most evident. Due to the lack of alternative evidence, we assumed identical relapse probabilities for both drugs. These aspects should be considered when interpreting our results.

Additionally, our model relies on international cost data (Spain) extracted from literature, which are applied to the German healthcare setting. This is only possible by assuming that in general, the standard of care of cUTI in Spain is comparable to Germany and differs only by the different price levels [19, 20], which may not fully capture the variability seen in real-world clinical settings. This may allow both an over- and underestimation for both scenarios cUTI with and without clinical relapse/readmission.

While the decision tree approach provides a transparent framework for health economic evaluation, it does not account for dynamic factors such as patient-specific characteristics or drug resistance patterns that may influence treatment outcomes. Due to the chosen perspective, no indirect costs (e.g. illness-related disability) were taken into account in our analysis, so we assume a conservative approach and the total costs for cUTI may be higher than shown in this study. Future research should aim to validate these findings with real-world healthcare data and incorporate more granular cost analyses preferably by a bottom-up cost data approach.

In conclusion, this study provides strong evidence supporting the health economic and clinical benefits of cefepime/enmetazobactam over piperacillin/tazobactam for the treatment of cUTI in Germany. By reducing relapse rates and associated healthcare costs, this novel antibiotic regimen represents a promising option for optimizing resource allocation and improving patient care outcomes.

## Conclusion

This study is the first to provide a health economic evaluation of cefepime/enmetazobactam in the treatment of cUTI from the German payers’ perspective. The use of cefepime/enmetazobactam for cUTI led to slightly reduced costs compared to piperacillin/tazobactam due to higher microbiological eradication probabilities and consequently lower relapse/readmission rates on a case level but might lead to substantially reduced costs from a population level. Further research on cUTI and its treatment options or another perspective for health economic evaluation is needed to gain deeper insights.

## Conflict of interests

J.R., S.M.W.H., D.S., A.C.F., M.S.K., and F.K. are independent employees of VITIS Healthcare Group and received research funding from Advanz Pharma Germany GmbH. S.M.W.H. has received research and travel grants from Astellas, Merck, and Tillotts; research grants from Basilea, Gilead, and 3 M; travel grants from Pfizer; and lecture honoraria from Astellas, Merck, and Tillotts. O.W. has received research grants, speaker fees or travel grants from Amgen, Alexion, Astellas, AstraZeneca, Basilea, Biotest, Bristol-Myers Squibb, Correvio, Chiesi, Gilead, GSK, Hexal, Janssen, Dr. F. Köhler Chemie, MSD, Novartis, Roche, Pfizer, Sanofi, TEVA and UCB. O. Witzke is a member of the DGfN, DTG, RWGIM, DGMI and a member of the executive board of the DGI. M.J.G.T.V. has received speaker fees from Merck/MSD, 3 M, Ferring, Astellas, Uniklinik Karlsruhe, Uniklinik Köln, Akademie für Infektionsmedizin, Klinikum Essen, Pfizer, Universitätsklinikum Heidelberg, Uniklinik Frankfurt, Landesärztekammer Hessen, Janssen, Institute Merieux, Forum für medizinische Fortbildung GmbH, Universitätsklinikum Freiburg, Berliner Dialyseminar, ADKA, Falk Foundation, St Johannes Hospital, DiaLog Service, CED Service, Ärztekammer Niedersachsen, St Josef Hospital, Limbach Gruppe SE, SUMIT OXFORD Ltd, EUMEDICA, KIT Kongress, Tillotts Pharma, Helios Kliniken, Lahn-Dill Kliniken, Gilead, and Klinikum Leverkusen. O.A.C. has received grants or contracts from iMi, iHi, DFG, BMBF, Cidara, DZIF, EU-DG RTD, F2G, Gilead, Med-Pace, MSD, Mundipharma, Octapharma, Pfizer and Scynexis, received consulting fees from Abbvie, AiCuris, Basilea, Biocon, Cidara, Seqirus, Gilead, GSK, IQVIA, Janssen, Matinas, MedPace, Menarini, Molecular Partners, MSG-ERC, Mundipharma, Noxxon, Octapharma, Pardes, Partner Therapeutics, Pfizer, PSI, Scynexis, Seres, Elion Therapeutics and Melinta, received payment or honoraria from Abbott, Abbvie, Akademie für Infektionsmedizin, Al-Jazeera Pharmaceuticals/Hikma, amedes, AstraZeneca, Deutscher Ärzteverlag, Gilead, GSK, Grupo Biotoscana/United Medical/Knight, Ipsen Pharma, Medscape/WebMD, MedUpdate, MSD, Moderna, Mundipharma, Noscendo, Paul-Martini-Stiftung, Pfizer, Sandoz, Seqirus, Shionogi, streamedup!, Touch Independent and Vitis, received payment for expert testimony from Cidara, and participated on a Data Safety Monitoring Board or Advisory Board for Boston Strategic Partners, Cidara, IQVIA, Janssen, MedPace, PSI, Pulmocide, Shionogi, The Prime Meridian Group, Vedanta Biosciences, AstraZeneca and Melinta.

## Ethics approval

Ethics approval is not required for this study in accordance with local or national guidelines.

## Supplementary Information

Below is the link to the electronic supplementary material.

Supplementary file1 (DOCX 274 KB)

## Data Availability

All data is publicly available and does not include individual patient cost data.
