# Supplementary material for: Evaluating the health economic impact of cefepime/enmetazobactam in complicated urinary tract infections in the German setting: a cost analysis from payer perspective
Source: Infection. 2025 Dec 26;54(2):807–16. doi: 10.1007/s15010-025-02711-9 (PMC13021750; doi:10.1007/s15010-025-02711-9)
Supplement: Supplementary file 1 — Supplementary file1 (DOCX 274 KB) [file 15010_2025_2711_MOESM1_ESM.docx]

# Supplementary material

Supplementary Table 7 Calculations for Figure 1 Decision tree.

| I | €5,212.78 = (6.2% $*$ €6,357.14) + (93.8% $*$€5,137.14) |
| --- | --- |
| II | €5,462.88 = (26.7% $*$ €6,357.14) + (73.3% $*$€5,137.14) |
| IIIa | €5,322.12 = (79.1% $*$ €5,121.78) + (13.4% $*$ €5,462.88) + (7.5% $*$ €6,357.14) |
| IIIb | €5,414.83 = (58.9% $*$ €5,121.78) + (30.0% $*$ €5,462.88) + (11.1% $*$ €6,357.14) |

Supplementary Table 8 Key for ICD codes (18).

| A41.0 | Sepsis caused by Staphylococcus aureus |
| --- | --- |
| A41.1 | Sepsis caused by other specified staphylococci |
| A41.2 | Sepsis caused by other non-specified staphylococci |
| A41.51 | Sepsis caused by other gram-negative pathogens: Escherichia coli [E. coli] |
| A41.52 | Sepsis caused by other gram-negative pathogens: Pseudomonas |
| A41.58 | Sepsis caused by other gram-negative pathogens: other gramnegative pathogens |
| A41.8 | Sepsis caused by other gram-negative pathogens: Escherichia coli [E. coli] |
| A41.9 | Other specified sepsis |
| N10 | Acute tubulointerstitial nephritis |
| N13.0 | Hydronephrosis caused by ureteropelvic obstruction |
| N13.1 | Hydronephrosis caused by ureteral stricture not elsewhere classified |
| N13.20 | Hydronephrosis due to obstruction caused by kidney stone |
| N13.21 | Hydronephrosis in obstruction caused by ureteral calculi |
| N13.29 | Hydronephrosis in obstruction caused by kidney and ureteral stone stone localisation unspecified |
| N13.3 | Other and unspecified hydronephrosis |
| N13.4 | Hydroureter |
| N13.5 | Kinking and stricture of the ureter without hydronephrosis |
| N13.60 | Hydronephrosis in ureteropelvic obstruction with infection of the kidney |
| N13.61 | Hydronephrosis caused by ureteral stricture not elsewhere classified with infection of the kidney |
| N13.62 | Hydronephrosis caused by obstruction due to kidney stone with infection of the kidney |
| N13.63 | Hydronephrosis caused by obstruction due to ureteral calculus with infection of the kidney |
| N13.64 | Hydronephrosis caused by obstruction due to kidney and ureteral stone, stone localisation unspecified, with infection of the kidney |
| N13.65 | Other and unspecified hydronephrosis with infection of kidney |
| N13.66 | Hydroureter with infection of kidney |
| N13.67 | Kinking and stricture of ureter without hydronephrosis with infection of kidney |
| N13.68 | Other and unspecified pyonephrosis |
| N13.7 | Uropathy in connection with vesicoureteral reflux |
| N13.8 | Other obstructive uropathy and reflux uropathy |
| N13.9 | Obstructive uropathy and reflux uropathy, not further specified |
| N17.01 | Acute renal failure with tubular necrosis, stadium 1 |
| N17.02 | Acute renal failure with tubular necrosis, stadium 2 |
| N17.03 | Acute renal failure with tubular necrosis, stadium 3 |
| N17.09 | Acute renal failure with tubular necrosis, not further specified |
| N17.11 | Acute renal failure with cortical necrosis, stadium 1 |
| N17.12 | Acute renal failure with cortical necrosis, stadium 2 |
| N17.13 | Acute renal failure with cortical necrosis, stadium 3 |
| N17.19 | Acute renal failure with cortical necrosis, not further specified |
| N17.21 | Acute renal failure with medullary necrosis, stadium 1 |
| N17.22 | Acute renal failure with medullary necrosis, stadium 2 |
| N17.23 | Acute renal failure with medullary necrosis, stadium 3 |
| N17.29 | Acute renal failure with medullary necrosis, not further specified |
| N17.81 | Other acute renal failure, stadium 1 |
| N17.82 | Other acute renal failure, stadium 2 |
| N17.83 | Other acute renal failure, stadium 3 |
| N17.89 | Other acute renal failure, not further specified |
| N17.91 | Acute renal failure, not further specified, stadium 1 |
| N17.92 | Acute renal failure, not further specified, stadium 2 |
| N17.93 | Acute renal failure, not further specified, stadium 3 |
| N17.99 | Acute renal failure, not further specified, stadium not further specified |
| N30.3 | Sepsis, not further specified |
| N30.0 | Acute cystitis |
| N30.1 | Interstitial cystitis (chronic) |
| N30.2 | Trigonum cystitis |
| N30.8 | Other cystitis |
| N30.9 | Cystitis, not further specified |
| N39.0 | Urinary tract infection, localisation unspecified |
| N39.88 | Other specified diseases of the urinary system |
| R10.2 | Pelvic and perineal pain |
| R10.3 | Pain localised in other parts of the lower abdomen |
| R11 | Nausea and vomiting |
| R33 | Urinary retention |
| T83.5 | Infection and inflammatory reaction caused by prosthesis implant or transplant in the urinary tract |

Supplementary Table 9 Key for OPS codes (19).

| 8-987.00 | Complex treatment for colonisation or infection with multidrug-resistant pathogens [MDR]: Complex treatment in a special isolation unit, up to 6 treatment days |
| --- | --- |
| 8-987.01 | Complex treatment for colonisation or infection with multidrug-resistant pathogens [MDR]: Complex treatment in a special isolation unit, minimum 7 to maximum 13 treatment days |
| 8-987.02 | Complex treatment for colonisation or infection with multidrug-resistant pathogens [MDR]: Complex treatment in a special isolation unit, minimum 14 to maximum 20 treatment days |
| 8-987.03 | Complex treatment for colonisation or infection with multidrug-resistant pathogens [MDR]: Complex treatment in a special isolation unit, minimum 21 treatment days |
| 8-987.10 | Complex treatment for colonisation or infection with multidrug-resistant pathogens [MDR]: Complex treatment in no special isolation unit, up to 6 treatment days |
| 8-987.11 | Complex treatment for colonisation or infection with multidrug-resistant pathogens [MDR]: Complex treatment in no special isolation unit, minimum 7 to maximum 13 treatment days |
| 8-987.12 | Complex treatment for colonisation or infection with multidrug-resistant pathogens [MDR]: Complex treatment in no special isolation unit, minimum 14 to maximum 20 treatment days |
| 8-987.13 | Complex treatment for colonisation or infection with multidrug-resistant pathogens [MDR]: Complex treatment in no special isolation unit, minimum 21 treatment days |

Supplementary Table 10 Queries for InEK DataBrowser (17).

| No. | Main diagnosis | Secondary diagnosis I | Secondary diagnosis II | OPS |
| --- | --- | --- | --- | --- |
| 1 | N39.0 | N17.01, N17.02, N17.03, N17.09, N17.11, N17.12, N17.13, N17.19, N17.21, N17.22, N17.23, N17.29, N17.81, N17.82, N17.83, N17.89, N17.91, N17.92, N17.93, N17.99 | A41.0, A41.1, A41.2, A41.51, A41.52, A41.58, A41.8, A41.9 | 8-987.00, 8-987.01, 8-987.02, 8-987.03,  8-987.10, 8-987.11, 8-987.12, 8-987.13 |
| 2 | N39.0 | N17.01, N17.02, N17.03, N17.09, N17.11, N17.12, N17.13, N17.19, N17.21, N17.22, N17.23, N17.29, N17.81, N17.82, N17.83, N17.89, N17.91, N17.92, N17.93, N17.99 | R10.2, R10.3 | 8-987.00, 8-987.01, 8-987.02, 8-987.03,  8-987.10, 8-987.11, 8-987.12, 8-987.13 |
| 3 | N39.0 | N17.01, N17.02, N17.03, N17.09, N17.11, N17.12, N17.13, N17.19, N17.21, N17.22, N17.23, N17.29, N17.81, N17.82, N17.83, N17.89, N17.91, N17.92, N17.93, N17.99 | T83.5 | 8-987.00, 8-987.01, 8-987.02, 8-987.03,  8-987.10, 8-987.11, 8-987.12, 8-987.13 |
| 4 | N39.0 | N17.01, N17.02, N17.03, N17.09, N17.11, N17.12, N17.13, N17.19, N17.21, N17.22, N17.23, N17.29, N17.81, N17.82, N17.83, N17.89, N17.91, N17.92, N17.93, N17.99 | R11 | 8-987.00, 8-987.01, 8-987.02, 8-987.03,  8-987.10, 8-987.11, 8-987.12, 8-987.13 |
| 5 | N39.0 | A41.0, A41.1, A41.2, A41.51, A41.52, A41.58, A41.8, A41.9 | R10.2, R10.3 | 8-987.00, 8-987.01, 8-987.02, 8-987.03,  8-987.10, 8-987.11, 8-987.12, 8-987.13 |
| 6 | N39.0 | A41.0, A41.1, A41.2, A41.51, A41.52, A41.58, A41.8, A41.9 | T83.5 | 8-987.00, 8-987.01, 8-987.02, 8-987.03,  8-987.10, 8-987.11, 8-987.12, 8-987.13 |
| 7 | N39.0 | A41.0, A41.1, A41.2, A41.51, A41.52, A41.58, A41.8, A41.9 | R11 | 8-987.00, 8-987.01, 8-987.02, 8-987.03,  8-987.10, 8-987.11, 8-987.12, 8-987.13 |
| 8 | N39.0 | R10.2 / R10.3 | T83.5 | 8-987.00, 8-987.01, 8-987.02, 8-987.03,  8-987.10, 8-987.11, 8-987.12, 8-987.13 |
| 9 | N39.0 | R10.2 / R10.3 | R11 | 8-987.00, 8-987.01, 8-987.02, 8-987.03,  8-987.10, 8-987.11, 8-987.12, 8-987.13 |
| 10 | N39.0 | T83.5 | R11 | 8-987.00, 8-987.01, 8-987.02, 8-987.03,  8-987.10, 8-987.11, 8-987.12, 8-987.13 |
| 11 | N39.0 | R33 | N17.01, N17.02, N17.03, N17.09, N17.11, N17.12, N17.13, N17.19, N17.21, N17.22, N17.23, N17.29, N17.81, N17.82, N17.83, N17.89, N17.91, N17.92, N17.93, N17.99 | 8-987.00, 8-987.01, 8-987.02, 8-987.03,  8-987.10, 8-987.11, 8-987.12, 8-987.13 |
| 12 | N39.0 | R33 | A41.0, A41.1, A41.2, A41.51, A41.52, A41.58, A41.8, A41.9 | 8-987.00, 8-987.01, 8-987.02, 8-987.03,  8-987.10, 8-987.11, 8-987.12, 8-987.13 |
| 13 | N39.0 | R33 | R10.2, R10.3 | 8-987.00, 8-987.01, 8-987.02, 8-987.03,  8-987.10, 8-987.11, 8-987.12, 8-987.13 |
| 14 | N39.0 | R33 | T83.5 | 8-987.00, 8-987.01, 8-987.02, 8-987.03,  8-987.10, 8-987.11, 8-987.12, 8-987.13 |
| 15 | N39.0 | R33 | R11 | 8-987.00, 8-987.01, 8-987.02, 8-987.03,  8-987.10, 8-987.11, 8-987.12, 8-987.13 |
| 16 | N39.0 | N13.0, N13.1, N13.20, N13.21, N13.29, N13.3, N13.4, N13.60, N13.61, N13.62, N13.63, N13.64, N13.65, N13.66, N13.67, N13.68, N13.7, N13.8, N13.9 | N17.01, N17.02, N17.03, N17.09, N17.11, N17.12, N17.13, N17.19, N17.21, N17.22, N17.23, N17.29, N17.81, N17.82, N17.83, N17.89, N17.91, N17.92, N17.93, N17.99 | 8-987.00, 8-987.01, 8-987.02, 8-987.03,  8-987.10, 8-987.11, 8-987.12, 8-987.13 |
| 17 | N39.0 | N13.0, N13.1, N13.20, N13.21, N13.29, N13.3, N13.4, N13.60, N13.61, N13.62, N13.63, N13.64, N13.65, N13.66, N13.67, N13.68, N13.7, N13.8, N13.9 | A41.0, A41.1, A41.2, A41.51, A41.52, A41.58, A41.8, A41.9 | 8-987.00, 8-987.01, 8-987.02, 8-987.03,  8-987.10, 8-987.11, 8-987.12, 8-987.13 |
| 18 | N39.0 | N13.0, N13.1, N13.20, N13.21, N13.29, N13.3, N13.4, N13.60, N13.61, N13.62, N13.63, N13.64, N13.65, N13.66, N13.67, N13.68, N13.7, N13.8, N13.9 | R10.2 / R10.3 | 8-987.00, 8-987.01, 8-987.02, 8-987.03,  8-987.10, 8-987.11, 8-987.12, 8-987.13 |
| 19 | N39.0 | N13.0, N13.1, N13.20, N13.21, N13.29, N13.3, N13.4, N13.60, N13.61, N13.62, N13.63, N13.64, N13.65, N13.66, N13.67, N13.68, N13.7, N13.8, N13.9 | T83.5 | 8-987.00, 8-987.01, 8-987.02, 8-987.03,  8-987.10, 8-987.11, 8-987.12, 8-987.13 |
| 20 | N39.0 | N13.0, N13.1, N13.20, N13.21, N13.29, N13.3, N13.4, N13.60, N13.61, N13.62, N13.63, N13.64, N13.65, N13.66, N13.67, N13.68, N13.7, N13.8, N13.9 | R11 | 8-987.00, 8-987.01, 8-987.02, 8-987.03,  8-987.10, 8-987.11, 8-987.12, 8-987.13 |
| 21 | N39.0 | N10, N39.88 | N17.01, N17.02, N17.03, N17.09, N17.11, N17.12, N17.13, N17.19, N17.21, N17.22, N17.23, N17.29, N17.81, N17.82, N17.83, N17.89, N17.91, N17.92, N17.93, N17.99 | 8-987.00, 8-987.01, 8-987.02, 8-987.03,  8-987.10, 8-987.11, 8-987.12, 8-987.13 |
| 22 | N39.0 | N10, N39.88 | A41.0, A41.1, A41.2, A41.51, A41.52, A41.58, A41.8, A41.9 | 8-987.00, 8-987.01, 8-987.02, 8-987.03,  8-987.10, 8-987.11, 8-987.12, 8-987.13 |
| 23 | N39.0 | N10, N39.88 | R10.2, R10.3 | 8-987.00, 8-987.01, 8-987.02, 8-987.03,  8-987.10, 8-987.11, 8-987.12, 8-987.13 |
| 24 | N39.0 | N10, N39.88 | T83.5 | 8-987.00, 8-987.01, 8-987.02, 8-987.03,  8-987.10, 8-987.11, 8-987.12, 8-987.13 |
| 25 | N39.0 | N10, N39.88 | R11 | 8-987.00, 8-987.01, 8-987.02, 8-987.03,  8-987.10, 8-987.11, 8-987.12, 8-987.13 |
| 26 | N39.0 | A41.0, A41.1, A41.2, A41.51, A41.52, A41.58, A41.8, A41.9, N10,  N13.0, N13.1, N13.20, N13.21, N13.29, N13.3, N13.4, N13.60, N13.61, N13.62, N13.63, N13.64, N13.65, N13.66, N13.67, N13.68, N13.7, N13.8, N13.9,  N17.01, N17.02, N17.03, N17.09, N17.11, N17.12, N17.13, N17.19, N17.21, N17.22, N17.23, N17.29, N17.81, N17.82, N17.83, N17.89, N17.91, N17.92, N17.93, N17.99,  N30.0, N30.1, N30.2, N30.3, N30.8, N30.9,  N39.88,  R10.2, R10.3,  R11,  R33,  T83.5 | U81.40, U81.41, U81.42, U81.43, U81.44, U81.45, U81.46, U81.47, U81.48, U81.50, U81.51 | -/- |

Table 11 Probabilities for decision-tree sensitivity analysis.

|  | Clinical cure AND microbiological eradication | | Clinical cure AND microbiological persistence | | NO clinical cure AND microbiological persistence | |
| --- | --- | --- | --- | --- | --- | --- |
| Cefepime/ enmetazobactam | 79.1% | | 13.4% | | 7.5% | |
| Piperacillin/ tazobactam | 58.9% | | 30.0% | | 11.1% | |
|  | 5.1% | 94.9% | 17.5% | 72.5% | 100% | 0% |
|  | Clinical relapse | No relapse/ readmission | Clinical relapse | No relapse/ readmission | Clinical relapse | No relapse/ readmission |


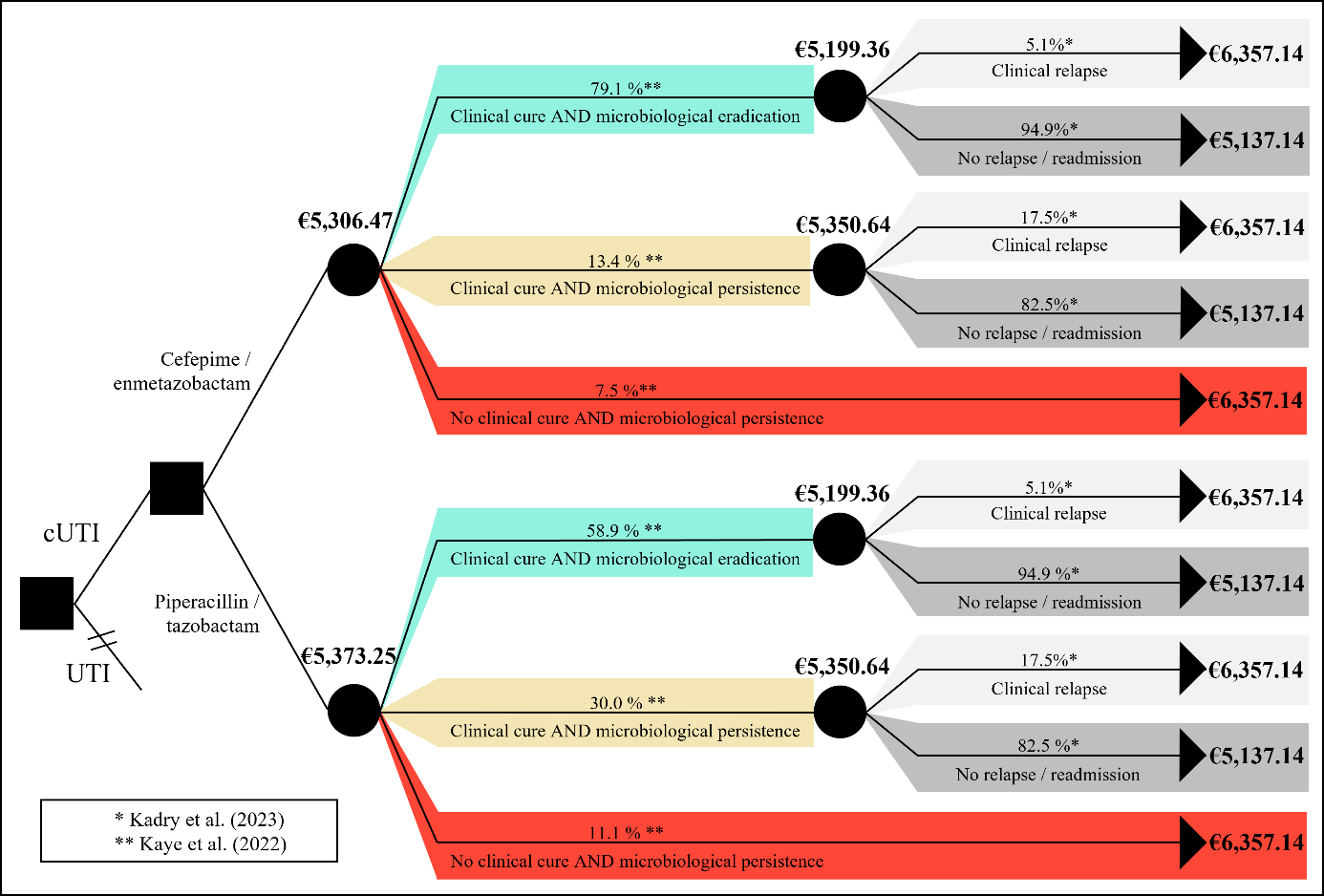


Supplementary Figure 2 Decision tree sensitivity analysis.

Supplementary Table 12 Calculations for Figure 2 Decision tree sensitivity analysis.

| I | €5,199.36 = (5.1% $*$ €6,357.14) + (94.9% $*$€5,137.14) |
| --- | --- |
| II | €5,350.64 = (17.5% $*$ €6,357.14) + (82.5% $*$€5,137.14) |
| IIIa | €5,306.47 = (79.1% $*$ €5,199.36) + (13.4% $*$ €5,350.64) + (7.5% $*$ €6,357.14) |
| IIIb | €5,373.25 = (58.9% $*$ €5,199.36) + (30.0% $*$ €5,350.63) + (11.1% $*$ €6,357.14) |
